# A Case of Tubercular Tumour of the Cerebellum

**Published:** 1908-06-13

**Authors:** Arthur G. Dunn

**Affiliations:** Resident Medical Officer to Fleming Hospital for Children, Newcastle-on-Tyne.


### Resident Medical Officers' Department.

### A CASE OF TUBERCULAR TUMOUR OF THE CEREBELLUM.

By ARTHUR G. DUNN, M.B., B.S., Durham, M.R.C.S., L.R.C.P., Resident Medical Officer to Fleming Hospital for Children, Newcastle-on-Tyne.

The following case presents features of considerable interest, being by no means typical either in the mode of onset or course of its symptoms. It is that of a child, T. J., aged two and a half years, who was admitted to the above hospital on July 4, 1907, under the care of Dr. McCracken, as a case of tetany. The history dated back to some three months previously, during which period he was stated to have been walking badly, and to have had frequent attacks of giddiness. During the three weeks previous to admission he had intermittent attacks of rigidity of the arms and legs, lasting for some hours. He had had no previous illness of importance, and the family history was good.

On admission he was noted to be a well-nourished child, but presenting well-marked osseous signs of rickets, such as large, square, head, open anterior fontanelle, rosary of ribs, and enlarged epiphyses. The temperature was normal. He was in an extremely irritable condition, screaming loudly when approached, so that satisfactory examination of the chest was well-nigh impossible. The limbs were in a position characteristic of tetany, there being flexion of the arms at the elbows, and of the hands upon the wrists, the thumbs being pressed into the palms. The lower limbs were rigidly extended at the knees, and there was plantar flexion of the feet. The knee-jerks were active and the plantar reflexes were present. It was noted that the hands were extremely tremulous when an attempt was made to grasp any object. On July 5, the day following admission, the spasm of the limbs had completely passed off, but he was still very irritable. He remained in this condition for some days, having occasional spasms, and sometimes a rise of temperature to 100° F. On July 19 and 20 he vomited two or three times. On the 25th he was obviously much worse. The temperature went up to 101° F. The right arm was found to be paretic, and the left, though rigid, was very tremulous. The legs were still rigid as in previous spasms. The muscles at the back of the neck were also found to be rigid, and there was bulging of the anterior fontanelle. With considerable difficulty an examination of the eyes was made, and early optic neuritis was found in each eye, with a flame-shaped hæmorrhage just below the left disc. On July 26 lumbar puncture was performed, and a pressure of 12 inches was registered in the manometer, going up to 16 inches when the child screamed. The fluid was quite clear. A diagnosis of meningitis was now made, probably tuberculous, an opinion sustained by the discovery of a tubercular focus at the apex of the right lung a little later. Lumbar punctures were made on two subsequent occasions, and several drachms of clear fluid withdrawn for bacteriological examination: the latter, however, revealed nothing. Sensibility was maintained during this time, as the child could be made to laugh, and take his food well. During the month of August, however, he ran a persistently high temperature, and steady wasting set in, death ensuing on August 28. The necropsy revealed tuberculous foci at the apices of both lungs, particularly the right, with tubercles on the pleuræ. A few small tubercular glands were found in the mesentery. The cerebrum was very soft and œdematous. There was clear fluid present at the base of the brain. On examining the pia mater at the base of the brain a very few tubercles were found lying along the Sylvian fissures. There was great distension of the lateral ventricles with fluid, the foramen of Munro being very large. On cutting into the cerebellum a large tubercular mass was found in the right lateral lobe and a similar tumour in the vermis, each about the size of a walnut.

In Taylor's text-book of medicine a group of symptoms is mentioned as characteristic of tumour of the middle lobe of the cerebellum which corresponds in many ways with the peculiar combination described in the above case. For instance, he mentions the following symptoms as occurring in tumour of the middle lobe: Headache aggravated by movement, ataxy, vertigo, oscillation of the eyeballs, retraction of the head, tetaniform convulsions, and tremors of the muscles of the limbs somewhat simulating those of disseminated sclerosis. As will be seen from an examination of the history and clinical course of this case, most of these symptoms were displayed, but one has to remember that the difficulties of localisation of a brain lesion in a child of two and a half years are very considerable, and the rarity of cerebellar lesions in particular in no wise diminishes these difficulties. Lastly, the simulation of simple tetany in the early history of the case is a point of considerable interest, while we might also point out how valuable information was gained from the ophthalmoscopic examination.

